# Small practice effects on neurocognitive testing – markers of early cognitive impairment among adolescents with SLE?

**DOI:** 10.1186/1546-0096-10-S1-A26

**Published:** 2012-07-13

**Authors:** Eyal Muscal, Debra L Canter, Douglas R Bloom, Barry L Myones, Stephen L Holliday, Robin L Brey

**Affiliations:** 1 Baylor College of Medicine, Houston, TX, USA; 2 University of Texas Health Science Center, San Antonio, San Antonio, TX, USA; 3 US Dept. of Veterans Affairs, San Antonio, TX, USA

## Purpose

Neurocognitive impairment is a prevalent feature of SLE. There is a paucity of data on the longitudinal assessment of cognitive performance of pediatric patients. There is no consensus on the timing of repeated neuropsychological evaluation in this population.

### Objective

To describe longitudinal changes for both a traditional neuropsychological battery (TNPB) and the computerized Pediatric-Automated Neuropsychological Assessment Metrics (Ped-ANAM) among adolescents with SLE. We hypothesized there would be improvement in scores typical of practice effects among patients with minimal disease activity.

## Methods

TNPB, Ped-ANAM, quality of life (PedsQL), and depression scales (CDI-S) were administered by psychology associates blinded to clinical status at two research visits. Differences in mean TNPB standardized scores, Ped-ANAM throughput scores (raw Throughput=correct responses/minute), and clinical variables between visits were compared using paired Student t-tests. Multi-variable linear regression analysis was used to assess the influence of initial test performance, aPL/LAC status, disease duration, and cumulative prednisone dosing on composite TNPB or Ped-ANAM scores at a second visit.

## Results

Twenty adolescents without active NPSLE were evaluated at two time points (mean of 6.2 + 1.8 months apart). Mean age at first visit was 15.8 + 1.4 years (range 13-18; 95% female, 50% Hispanic, 25% African-American). Disease duration at entry was 22.5 + 12.6 months. Only 2 patients (10%) had a NPSLE history prior to study entry, and 3 (15%) a history of biopsy-proven nephritis. Cerebral volume loss was observed on 3T anatomic MRIs of 12 subjects (60%). SLEDAI and SLICC scores were low and not statistically different between visits (< 4 and < 1 respectively). A history of LAC or aPL positivity was present in 8 (40%) and 16 (80%), respectively, at study entry. Full scale IQ scores were within average range (102.0 + 9.0 points), and unchanged between visits. Mean depression and quality of life indices scores did not reflect distress at either visit. The lowest mean TNPB scores at both visits were on tests of verbal fluency and visual-motor integration. There were small improvements in the majority of individual TNPB and Ped-ANAM scores (Z score mean increase 0.19 + 0.14 for TNPB and 0.48 + 0.41 for Ped-ANAM tests). TNPB test score improvements were often smaller than those described in normative population validation studies. Significant improvements were observed on the Ped-ANAM subscales of Code Substitution-Delayed (Z change=0.63, p=0.02) Logical Relation (Z score change=0.71, p<0.01) and Spatial Processing (Z change=1.3, p<0.01). Significant decrements were observed on the Trail-Making Test B (Z=-0.8, p<0.05)] and two Ped-ANAM tests [Math Processing Z=-0.77, p<0.01 and Procedural Reaction Z=-1.0, p<0.01]. Visit 1 composite NP scores were the only significant predictors of visit 2 composite scores in multivariable regression analyses (TNPB β=0.44, p=0.03 and Ped-ANAM β=0.62, p<0.01).

## Conclusion

Smaller than expected practice effects may reflect impaired learning ability early in the disease course of adolescents with SLE. Replication with larger controlled samples, and longer follow-up is planned.

## Disclosure

Eyal Muscal: None; Debra L. Canter: None; Douglas R. Bloom: None; Barry L. Myones: None; Stephen L. Holliday: None; Robin L. Brey: None.

